# Manipulating stomatal density enhances drought tolerance without deleterious effect on nutrient uptake

**DOI:** 10.1111/nph.13598

**Published:** 2015-08-13

**Authors:** Christopher Hepworth, Timothy Doheny‐Adams, Lee Hunt, Duncan D. Cameron, Julie E. Gray

**Affiliations:** ^1^ Department of Molecular Biology and BiotechnologyUniversity of SheffieldSheffieldS10 2TNUK; ^2^ Department of BiologyUniversity of YorkYorkYO10 5DDUK; ^3^ Department of Animal and Plant SciencesUniversity of SheffieldSheffieldS10 2TNUK

**Keywords:** *Arabidopsis thaliana*, drought tolerance, epidermal patterning factor (EPF), mass flow, nitrate, stomata, transpiration, water use efficiency

## Abstract

- Manipulation of stomatal density was investigated as a potential tool for enhancing drought tolerance or nutrient uptake.
- Drought tolerance and soil water retention were assessed using Arabidopsis epidermal patterning factor mutants manipulated to have increased or decreased stomatal density. Root nutrient uptake via mass flow was monitored under differing plant watering regimes using nitrogen‐15 (^15^N) isotope and mass spectrometry.
- Plants with less than half of their normal complement of stomata, and correspondingly reduced levels of transpiration, conserve soil moisture and are highly drought tolerant but show little or no reduction in shoot nitrogen concentrations especially when water availability is restricted. By contrast, plants with over twice the normal density of stomata have a greater capacity for nitrogen uptake, except when water availability is restricted.
- We demonstrate the possibility of producing plants with reduced transpiration which have increased drought tolerance, with little or no loss of nutrient uptake. We demonstrate that increasing transpiration can enhance nutrient uptake when water is plentiful.

## Introduction

A widespread solution to ensuring crop yields is the application of extensive irrigation (Rosegrant *et al*., 2009). However, with an increase in human population and decrease in clean water availability, irrigation is becoming a less viable solution. Future climate forecasts suggest an increased frequency of extreme weather events including more and longer lasting droughts, and water availability is expected to further threaten food security (Godfray *et al*., 2010; IPCC, 2013; Elliott *et al*., 2014). Although genetic manipulation and the screening of crop germplasms have yielded improvements in plant drought tolerance or water use efficiency, the development of corresponding crop varieties has been slow and findings have only on rare occasions been translated through to the field (Langridge & Reynolds, 2015).

The uptake of nutrients from the soil is inextricably linked to water uptake and movement in soils but to our knowledge, few studies have considered the nontarget effects of drought tolerance on such factors that may limit plant performance. Plants rapidly deplete nutrients from the rhizosphere, which are replenished via dissolved nutrients carried in the flow of water to plant roots by mass flow, a process driven by transpiration (*E*) (Barber, 1962). We are largely ignorant of the extent to which plant water requirements, and mass flow, can be reduced before detrimentally impacting on the nutrient status of the plant. Nitrogen (N) an important nutrient with regard to plant growth, moves through the soil primarily by mass flow. Consequently, it might be expected that drought tolerant crop varieties with low levels of *E*, would be more reliant on inputs of N fertilizer, and indeed several previous studies have demonstrated a correlation between *E* and N uptake for example (Shimono & Bunce, 2009). This problem could be further compounded as levels of *E* are expected to be decreased by future elevated atmospheric CO_2_ levels (Ainsworth & Rogers, 2007). Indeed, there is current debate over whether future CO_2_ levels will have an adverse effect on the uptake of nutrients important to the human diet. Recent meta‐analysis of free air CO_2_ enrichment experiments have linked elevated CO_2_ to reductions in nutrients including iron and zinc in C_3_ crops (Myers *et al*., 2014), with carbon dilution and/or reductions in mass flow suggested as possible explanations (Taub & Wang, 2008; McGrath & Lobell, 2013). Clearly, providing drought tolerance at the expense of crop yields and nutrient content is a nonsustainable strategy. These multiple constraints of water and nutrient availability, together with predicted climate instability pose a serious threat to food security (Poppy *et al*., 2014).

Over the past decade the signaling pathway that controls the formation of stomata (the microscopic leaf pores that control *E*) has become better understood (Casson & Gray, 2008; Lau & Bergmann, 2012; Pillitteri & Torii, 2012). This knowledge allows us to study the physiological implications of altering stomatal density (*D*) within plants of the same genetic background. Manipulating the levels of peptide signals known as epidermal patterning factors (EPFs), which act during leaf formation to regulate stomatal development, has produced *Arabidopsis thaliana* plant lines which have stomatal densities ranging from *c*. 20% to 325% of normal levels. Plants with low *D* have much reduced levels of *E* (Hunt & Gray, 2009; Doheny‐Adams *et al*., 2012; Tanaka *et al*., 2013), and are able to grow larger, especially under conditions of water restriction (Doheny‐Adams *et al*., 2012). Recently plants with reduced *D* have been shown to have significantly enhanced water use efficiency (Franks *et al*., 2015) suggesting that they may be better able to survive drought conditions. Here, using plants with altered EPF levels (Hara *et al*., 2007, 2009; Hunt & Gray, 2009) which have increased or decreased *D*, together with natural abundance stable isotope (carbon‐13, ^13^C) profiling and ^15^N isotope tracers; we show the impact of manipulating stomatal development on both drought tolerance and nutrient uptake.

## Materials and Methods

### Plant growth

*Arabidopsis thaliana* (L.) Heynh stomatal development mutant genotypes were created in Col‐0 background and have been previously described (Hunt & Gray, 2009). Col‐0 was used as the wild‐type control. Seeds were stratified (72 h at 4°C in dark) before transfer to an environmentally controlled growth chamber (Conviron model BDR16) at 22°C : 16°C, 9 h light, 200 μmol m^−2^ s^−1^, 15 h dark. Plants were grown in M3 compost/perlite (4 : 1) and watered every 3 d with 200 ml of water unless otherwise stated. For drought treatment, watering ceased when plants were 4 wk old. Water‐restricted growth conditions are described later.

### Stomatal density determination

Stomatal counts were taken from the abaxial surface of fully expanded leaves from mature leaf rosettes. Dental resin (Coltene, Whaledent, Switzerland) was applied and left to set before removing the leaf and applying clear nail vanish to the resin at the maximum leaf width. Stomatal counts were determined from nail varnish impressions by light microscopy (Olympus BX51; Tokyo, Japan) from images captured with digital microscope eyepiece (HiROCAM MA88‐300A; Shanghai, China). Five plants per genotype, three leaves per plant and three areas per leaf, were examined.

### Stomatal conductance and transpiration assays

**Figure 1:**
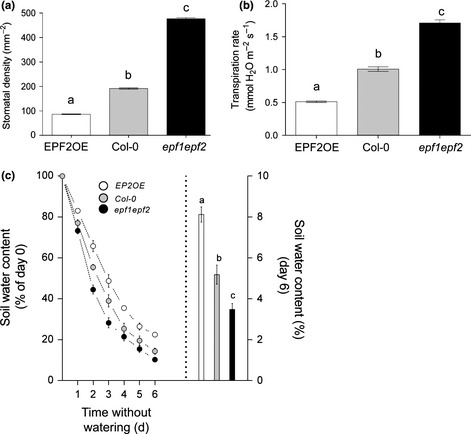
Manipulation of epidermal patterning factor (EPF) peptide levels leads to increased or decreased stomatal density, transpiration rate and soil drying. (a) Density of stomata on the abaxial surface of mature leaves of plants with increased or decreased levels of epidermal patterning factor 2 (*Arabidopsis thaliana EPF2* OE and *epf1epf2* genotypes) and Col‐0 wild‐type background control (*n* = 5). (b) Transpiration rates of stomatal density mutants and Col‐0 (*n* = 5). (c) Change in soil water content over a 6 d period of drought compared to water saturated soil at day 0 (left axis) and absolute percentage soil water content on day 6 of drought (right axis) (*n* = 8). Different letters indicate significant difference between means (*P* < 0.05; Tukey test after one‐way analysis of variance). Error bars, ± standard error.

**Figure 2:**
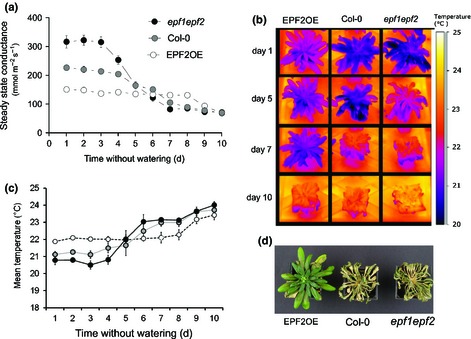
Reduced stomatal density improves drought tolerance in *Arabidopsis thaliana*. (a) Stomatal conductance across a 10‐d period of drought (*n* = 5). *EPF2* OE and *epf1epf2* conductances were both significantly different from Col‐0 at days 1–4, and *EPF2* OE values were significantly different from Col‐0 at days 5, 7 and 8 (*P* < 0.05; Tukey test after one‐way analysis of variance). Error bars, ± standard error. (b) False colored infrared‐thermal images of representative plants during drought under the same conditions as (a). (c) Mean leaf temperatures of plants during drought experiment. (d) Photographs of representative plants following 10 d of drought, 2 d after subsequent re‐watering.

For *E* measurements (Fig. 1b) a LI‐6400 portable photosynthesis system (LiCor, Lincoln, NE, USA) was used to carry out IRGA on mature leaves attached to the plants during the middle of the day that is, from 3 to 6 h into the photoperiod. Relative humidity of the chamber was kept at 65–75% using self‐indicating desiccant, flow rate was 500 μmol s^−1^ and block temperature 20°C. Carbon dioxide was maintained at 400 ppm, and light intensity at 300 μmol m^−2^ s^−1^. Plants were allowed to equilibrate for 20 min before measurements were taken every 30 s for 15 min with the IRGA being matched every 3 min. Five plants per genotype were analyzed. For daily monitoring of stomatal conductance on each day of drought (Fig. 2a) a calibrated Decagon Sc‐1 Porometer was used to enable analysis of all plants within the mid‐photoperiod. Three measurements were taken from three leaves of five plants per genotype. The same three leaves were examined on each day of the experiment. A FLIR SC660 thermal imaging camera was used to capture infra‐red images of plants within the growth chamber. Sixty infra‐red images of plants were taken over a period of 1 h, beginning 2 h after the start of the photoperiod. The first 20‐min period of image capture (after leaving the growth chamber) was omitted from the results to allow the growth chamber and plants to equilibrate. Five plants of each genotype were imaged and temperature data was recorded from the widest regions of three uncovered leaves per plant. Images were analyzed using ThermaCAM Researcher 2.9 Professional (FLIR Systems, Wilsonville, OR, USA) to calculate mean leaf temperature.

### Soil moisture level

Soil moisture readings were taken each day at mid‐photoperiod using a calibrated ML3 soil moisture probe from Delta T Devices (accuracy ± 1%). Mean values were calculated from readings from three measurements per pot from eight plants per genotype. Before the start of the experiment, growth medium (M3 compost) was homogenized, sieved and weighed to ensure equal amounts per pot. All pots were watered until saturated, stood in water for a further hour and excess water removed by blotting. Mean starting soil water content was calculated to be 38%. The mean difference in soil moisture between genotypes at the start of the experiment was < 1.25%.

### ^15^N and δ^13^C measurement

To assess nutrient uptake via mass flow a two pot system was used (Supporting Information Fig. S1). A 3‐wk‐old plant was placed in the ‘inner’ pot (with a volume of *c*. 500 cm^3^) which had large uniform windows cut out and covered with 10 μm mesh held in place by Tensol 12 glue (Bostik Ltd, Paris, France). When the plants were 4 wk old pots were placed inside ‘outer’ pots (with a volume of *c*. 1265 cm^3^), containing homogenously dampened, autoclaved compost. Five 1 ml injections of 4 mg ml^−1^ ammonium nitrate solution (supplied as 99% ^15^NH_4_^15^NO_3_ from Sigma‐Aldrich) were injected at predetermined points along the edge of the outer pot using a custom‐made nine side port needle. ‘Well‐watered’ plants were supplied with 50 ml of water into each outer pot every 3 d whilst ‘water‐restricted’ plants were given 20 ml of water. For both of the two watering regimes the inner pots were supplied with *c*. 10 ml of water every 3 d by misting with a spray bottle. Nitrogen‐15 concentration was analyzed from nine plants of each genotype and nine plants of Col‐0 wild‐type under each watering condition. Four plants of each genotype were watered in the same manner but not supplied with ^15^N and were used as blank controls for natural abundance of ^15^N. Three pots containing soil and no plants were injected with ^15^N and used as controls to measure any movement of ^15^N independent of plant water uptake, under each watering regime. Plants were propagated under these conditions for 3 wk. Positions were randomized after each watering event. After 3 wk, plants were harvested and ^15^N, ^13^C and ^12^C concentrations analyzed simultaneously by continuous‐flow isotope‐ratio mass spectrometry. Total ^15^N mg g^−1^ was calculated from shoot dry weight, N concentration and At% of natural abundance controls. The δ^13^C was assessed from three to five homogenized leaves from the top of the rosette which had developed under the two different watering regimes, converted to leaf carbon isotope discrimination (‰ leaf ∆^13^C) using previously described calculations and a measurement of growth chamber air of δ^13^C of −10.4‰ (Farquhar & Richards, 1984; Franks *et al*., 2015).

## Results

The *EPF2*OE mutant constitutively and ectopically over‐expresses the epidermal patterning factor EPF2, and in our experimental conditions had only 35% of the *D* of wild‐type on the abaxial surface of mature leaves. Conversely, the double mutant plants *epf1epf2*, which lack both EPF1 and EPF2, had 237% of wild‐type leaf *D* (Fig. 1a). We confirmed that the significant alteration in *D* in these plants is associated with changes in *E*. We found that in well‐watered conditions, plants with low *D* (*EPF2*OE) had a lower steady state level of *E* and plants with a high *D* (*epf1epf2*) had an elevated level of steady state *E* (50% and 170% of Col‐0, respectively; Fig. 1b) in line with our previous observations (Franks *et al*., 2015). Using a separate set of plants we then investigated whether changes in *D* could affect the rate of soil drying over a period of drought. Plants with low *D* had lower rates of soil drying and retained 8% rhizosphere water content after 6 d without watering. By contrast, high *D* plants had enhanced levels of soil drying and by day 6 had < 4% soil water content remaining within the rhizosphere (Fig. 1c).

To assess whether these alterations in leaf transpiration and soil water content could impact on drought tolerance, we conducted a terminal drought experiment. Measurements of stomatal conductance (*g*_s_) and infrared thermography images were recorded daily. As might be expected, this revealed a clear relationship between *D* and the dynamics of *g*_s_, *E* and surface leaf temperature (Fig. 2). Plants with reduced *D* were able to maintain a consistent level of *g*_*s*_ for 8 d without watering, until after 9 d of drought there was a drop in *g*_s_. In comparison, high *D* and wild‐type plants reacted much sooner to drought and their *g*_s_ dropped after 4 and 5 d without watering respectively (Fig. 2a). Differences in *E* across genotypes were also evident through alterations in leaf surface temperature (a proxy for evaporative cooling). This was evident at the start of the experiment when plants were well‐watered and throughout the drought period until 10 d without watering (Fig. 2b,c). After 10 d drought the plants were re‐watered. Only the *EPF2*OE low *D* plants recovered leaf turgor and continued to grow indicating that these plants with low *D* have increased drought tolerance (Fig. 2d).

**Figure 3:**
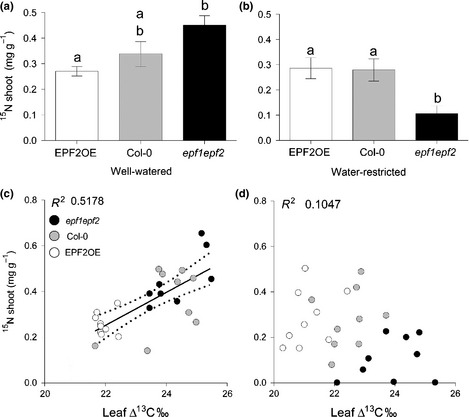
Effect of stomatal density on *Arabidopsis thaliana* shoot nitrogen concentrations. Nitrogen‐15 (^15^N) concentrations under; well‐watered (a) and water restricted conditions (b) (*n* = 9 for each watering treatment). (c) Linear regression between plant ^15^N and ∆^13^C measurement of water use efficiency in well‐watered conditions (*P* = 0.0002, *R* ^2^ = 0.5178) and (d) in water‐restricted conditions (*P* = 0.1326, *R* ^2^ = 0.1047). Different letters indicate significant difference between means (*P* < 0.05; Tukey test after one‐way analysis of variance). Dotted lines represent the 95% confidence band of the line of best fit. Error bars, ± standard error.

Having established that reducing *D* can improve drought tolerance we carried out a further experiment to investigate what consequence alterations in *D* and *g*_s_ could have on the rate of mass flow of dissolved nutrients to the roots. We consider it unlikely that either *EPF1* or *EPF2* gene manipulation would have a direct effect on nutrient uptake as neither gene is normally expressed in roots (Hara *et al*., 2007, 2009; Hunt & Gray, 2009). Nor do we consider it likely that soil microbes would affect N uptake since *Arabidopsis thaliana* is unable to form mycorrhizal associations. Our analysis of ^15^N concentrations in shoot material revealed that *D* influenced nutrient accumulation. High *D* plants (*epf1epf2*) took up a significantly greater amount of ^15^N than low *D* plants (*EPF2*OE) under well watered conditions (but neither level was significantly different from wild‐type) (Fig. 3a). Under restricted watering conditions when plant *g*_s_ and thus water loss was reduced in all three genotypes (Supporting Information Fig. S2) the pattern of N concentration was very different. High *D* plants acquired significantly less ^15^N in comparison to the other genotypes. Surprisingly, lack of water had no significant effect on the ^15^N concentrations of low *D* or wild‐type plants (Fig. 3a,b). We selected plants of similar sizes for this experiment and there were no significant differences between plant genotype shoot dry weights except that water‐restricted *epf1epf2* high *D* plants were 9% lighter than their watered‐restricted controls at the end of the experiment. Therefore at a per plant level, the pattern of ^15^N accumulation was similar to that shown in Fig. 3(a,b) and despite their smaller size, water‐restricted *epf1epf2* still accumulated significantly less ^15^N per plant than the other genotypes (not shown). Stable carbon isotopes levels were also analyzed. Plotting ^15^N concentration of individual plants against ∆^13^C values revealed a significant positive relationship between this proxy measure of water use efficiency and ^15^N concentration under well‐watered conditions (*R* ^2^ = 0.5178, *P* = 0.0002, Fig. 3c). However the significance of this relationship disappeared under a restricted watering regime (*R* ^2^ = 0.1047, *P* = 0.1326*,* Fig. 3d). These results indicate that under well‐watered conditions, an increase in *D* promotes ^15^N accumulation but at the expense of water use efficiency. Conversely, whether water availability was restricted or not, plants with low *D* exhibited improved water use efficiency without significant loss of ^15^N shoot concentration compared to wild‐type.

## Discussion

We conclude that by significantly reducing *D* through the manipulation of the expression level of an epidermal patterning factor, it is possible to reduce plant water use and to improve drought tolerance. Furthermore, although a trend towards decreased N concentrations was observed in well‐watered low *D* plants, drought tolerance was not accompanied by a significant reduction in foliar concentrations of ^15^N, neither when plants were well‐watered nor when water availability was restricted. Of course, it remains possible that the small difference between ^15^N concentrations in well‐watered low *D* plants and their controls could be statistically significant if a larger sample size were to be examined (we analyzed nine plants per genotype per treatment). Even so, it is unlikely that any reduction in nutrient uptake, significant or not, could be proportional to the 50% reduction in *E* in these low *D* plants. Thus, we propose that a reduction in *D* affects plant water loss to a disproportionally greater level than it affects nutrient uptake. Such an asymmetry between the magnitude of stomatal conductance and plant ^15^N concentration is not entirely unexpected. While mass flow of nutrients to the rhizosphere is driven by the extent of plant water loss via stomata, as our data show, the rate of uptake of nutrients is likely constrained by additional mechanisms. For example, root hair density, organic acid excretion as well as the expression of specific transporters may all define the nutrient uptake rate. It then follows that any mismatch in the magnitude of delivery and uptake, could generate the asymmetrical relationship between stomatal conductance and plant ^15^N concentration that we observed.

Despite their low levels of transpiration, under restricted watering conditions low *D* plants were able to maintain the same level of ^15^N per gram as when they were well‐watered. In addition to the earlier mechanisms, this may be explained at least in part, by a higher conservation of soil water content, which allowed the low *D* plants to maintain their ‘well‐watered’ rate of gas exchange for considerably longer than wild‐type plants when severe drought conditions were imposed. Indeed, a higher level of soil moisture has previously been reported to aid in the uptake of minerals including N (Van Vuuren *et al*., 1997). However, we found that increasing *D* could have a positive influence on nutrient concentrations under well‐watered conditions via the promotion of mass flow; a 70% increase in *E* resulted in an *c*. 33% increase in foliar ^15^N concentration in high *D* plants. However, enhanced nutrient uptake capacity came at the cost of diminished water use efficiency and greater susceptibility to drought. Furthermore, when high *D* plants were grown in water‐restricted conditions, nutrient concentration was reduced by 62%. Possible explanations for this large effect on nutrient accumulation when water was limited include a reduction in *g*_s_ through stomatal closure which in turn would reduce root conductivity and direct N uptake (Buljovcic & Engels, 2001). Thus a future strategy to promote nutrient uptake involving an increased level of transpiration, could be effective only under growth conditions where water availability is constant and plentiful.

We suggest that reductions in *D*, perhaps less severe than those of our experimental plants could provide a route towards improving drought tolerance, water use efficiency and soil water content without significantly affecting photosynthetic capacity (Franks *et al*., 2015) or nutrient accumulation by mass flow. The amount of water stored in soils is of fundamental importance to agriculture, with decreases in soil moisture expected to reduce global crop yields over the coming years (Long *et al*., 2006). Climate models predict that there are likely to be increases in soil drying and drought across many agricultural regions including the Mediterranean, southwest United States and southern African in the future, although enhanced precipitation may occur in other regions (IPCC, 2013). With this in mind, we believe that is worth noting that the positive effects of drought tolerance and soil water conservation that we report here, may be amplified if crop plants with low *D* were to be grown in monoculture.

## Supporting information

Please note: Wiley Blackwell are not responsible for the content or functionality of any supporting information supplied by the authors. Any queries (other than missing material) should be directed to the *New Phytologist* Central Office.

**Fig. S1** Diagram of the two pot experimental set‐up used to compare nutrient uptake by mass flow.
**Fig. S2** Growth in water‐restricted conditions reduces stomatal conductance.
