# Supplementary material for: Manipulating stomatal density enhances drought tolerance without deleterious effect on nutrient uptake
Source: New Phytol. 2015 Aug 13;208(2):336–41. doi: 10.1111/nph.13598 (PMC4973681; doi:10.1111/nph.13598)

New Phytologist

New Phytologist **Supporting Information**

Article title: **Manipulating stomatal density enhances drought tolerance without deleterious effect on nutrient uptake**
Authors: Christopher Hepworth, Timothy Doheny-Adams, Lee Hunt, Duncan D. Cameron and Julie E. Gray
Article acceptance date: 07 July 2015

The following Supporting Information is available for this article:

Fig. S1 Diagram of the two pot experimental set-up used to compare nutrient uptake by mass flow. ^{15}N-ammonium nitrate solution was injected into the outer plant pot medium outside a 10μm mesh and was able to move in solution with water into the inner pot medium containing the plant roots. Set-up used to compare isotope uptake between stomatal density mutants and Col-0 background.

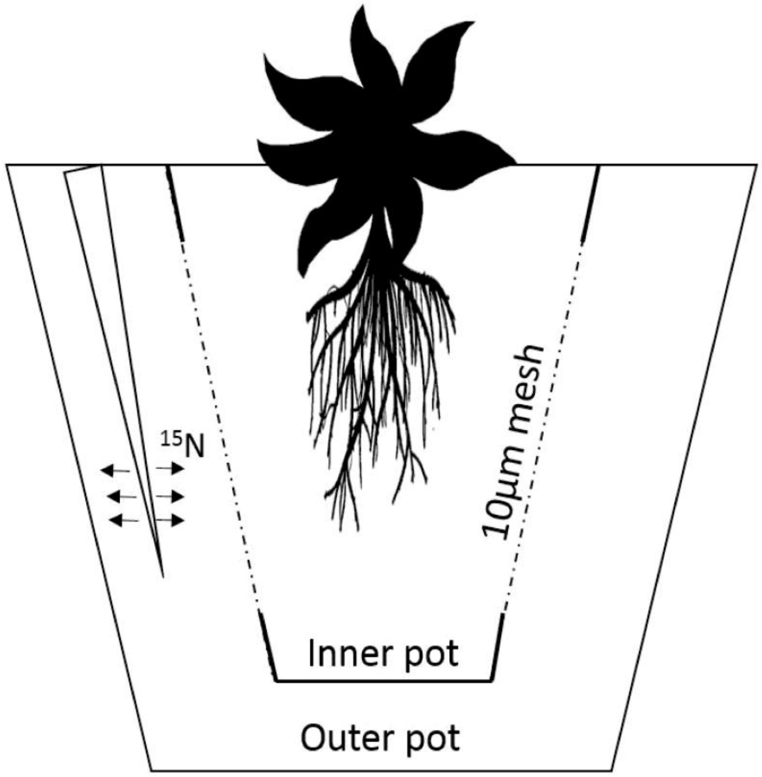

New Phytologist

Fig. S2 Growth in water-restricted conditions reduces stomatal conductance. Values expressed as a percentage of the conductance of well-watered plants of the same genotype grown under the same conditions except for water availability. Different letters indicate significant difference between means (P<0.05; Tukey test after one-way ANOVA). Error bars, ± SE.

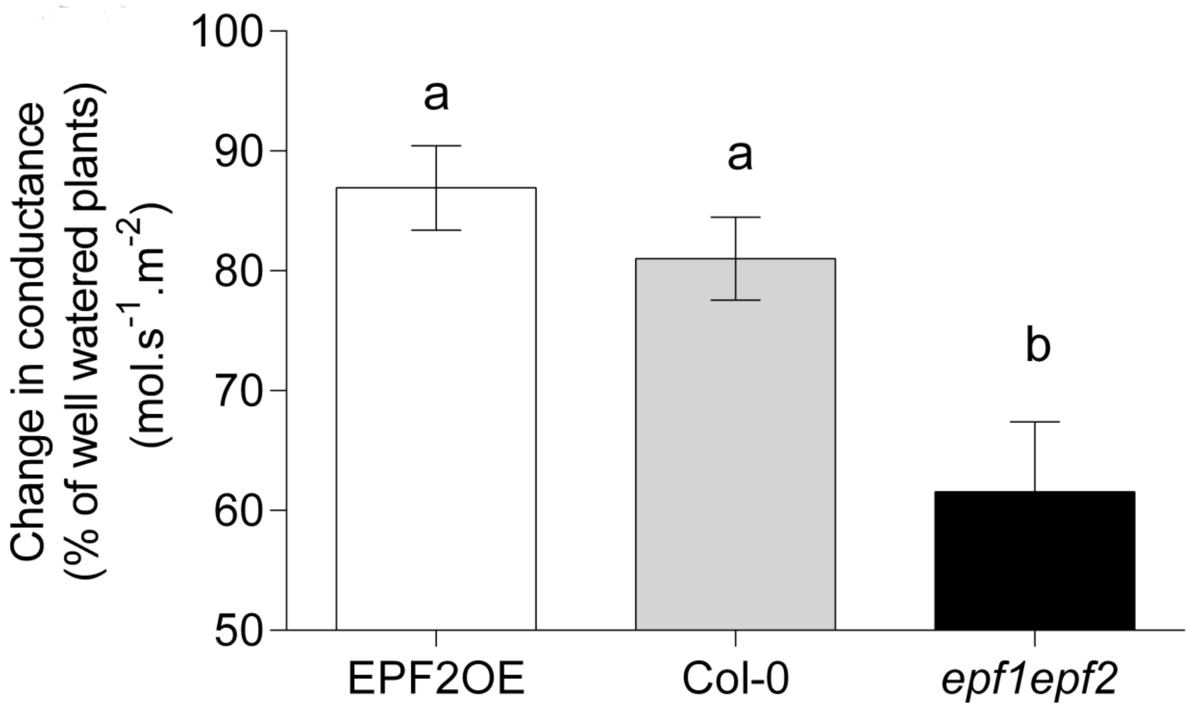

Supplement: Supplementary file 1 — Fig. S1 Diagram of the two pot experimental set‐up used to compare nutrient uptake by mass flow. Fig. S2 Growth in water‐restricted conditions reduces stomatal conductance. [file NPH-208-336-s001.pdf]
